# Potential co-infection of influenza A, influenza B, respiratory syncytial virus, and *Chlamydia pneumoniae*: a case report with literature review

**DOI:** 10.3389/fmed.2023.1325482

**Published:** 2024-01-08

**Authors:** Yuanyuan Feng, Shengzhu Wen, Song Xue, Meigui Hou, Ying Jin

**Affiliations:** Huangpu District Dapuqiao Community Health Center, Shanghai, China

**Keywords:** influenza, respiratory syncytial virus, *Chlamydia pneumoniae*, respiratory tract infection, co-infection

## Abstract

The occurrence of a co-infection involving four distinct respiratory pathogens could be underestimated. Here, we report the case of a 72-year-old woman who presented to a community hospital with a cough productive of sputum as her main clinical manifestation. Antibody detection of common respiratory pathogens revealed potential co-infection with influenza A, influenza B, respiratory syncytial virus, and *Chlamydia pneumoniae*. We treated her with 75 mg oseltamivir phosphate administered orally twice daily for 5 days, 0.5 g azithromycin administered orally for 5 days, and 0.3 g acetylcysteine aerosol inhaled twice daily for 3 days. The patient showed a favorable outcome on the eighth day after early diagnosis and treatment. Since co-infection with these four pathogens is rare, we performed an extensive PubMed search of similar cases and carried out a systematic review to analyze the epidemiology, clinical manifestations, transmission route, susceptible population, and outcomes of these four different pathogens. Our report highlights the importance for general practitioners to be vigilant about the possibility of mixed infections when a patient presents with respiratory symptoms. Although these symptoms may be mild, early diagnosis and timely treatment could improve outcomes. Additionally, further research is warranted to explore the potential influence of SARS-CoV-2 infection on the co-occurrence of multiple respiratory pathogens.

## 1 Introduction

Influenza has a short incubation period, rapid onset, and seasonal prevalence, and is associated with high mortality during pandemics, epidemics, and sporadic outbreaks. It can cause mild to severe illnesses in humans. Nearly 10 percent of the global population is affected by influenza each year and approximately half a million people perish annually (1). Human respiratory syncytial virus (RSV) is one of the main pathogens that cause upper and lower respiratory illnesses in the older population. RSV is a leading cause of morbidity and mortality among patients with severe lower respiratory tract infections, especially those who are older, vulnerable, immunocompromised, and have chronic cardiopulmonary disease (2–4). Globally, an estimated 14,000 in-hospital deaths were associated with RSV-related acute respiratory illnesses in 2015 (5). Therefore, RSV is increasingly recognized as a common cause of respiratory diseases in adults aged ≥ 65 years.

*Chlamydia pneumoniae* (Cpn) is an obligate intracellular pathogen and a common cause of human respiratory diseases, most commonly manifesting as pneumonia and bronchitis; it is responsible for 10% of community-acquired pneumonia cases and 5% of bronchitis, pharyngitis, and sinusitis cases (6).

Recently, a case of potential co-infection with influenza A, influenza B, RSV, and Cpn was treated at the outpatient department of our hospital. The co-infection of these four pathogens has rarely been reported. Here, we report the diagnosis and treatment process and perform a comprehensive review of the literature.

## 2 Case report

A 72-year-old woman presented to our general clinic on 27 March 2023, having suffered from coughing with sputum for 3 days. On the evening of 24 March 2023, the patient developed a clear cough producing large quantities of white, sticky sputum with mild shortness of breath and tachypnea. She did not have a fever, sore throat, chest pain, hemoptysis, dyspnea, abdominal pain, diarrhea, nausea, vomiting, muscle or joint aches, or any other symptoms. She reported no pre-existing diseases; she and her relatives had not left Shanghai, did not receive the influenza vaccine, had no history of alcohol or tobacco use, and had not taken other drugs at the time. Notably, the patient had previously been infected with the novel coronavirus 2019 (COVID-19) around 20 December 2022. During that previous infection, she experienced symptoms of fatigue and fever, with the highest recorded temperature being 38.9°C. Her condition improved within approximately 2 days of receiving oral acetaminophen (0.5 g twice daily) treatment, and her symptoms completely resolved after 3 days of intravenous administration of cefuroxime (1.5 g twice daily) in addition to ambroxol (30 mg twice daily).

Physical examination revealed a temperature of 36.8°C and an oxygen saturation of 98%. Coarse breathing sounds were detected in both lower lungs, with a small number of rales in the left lower lung. Her heart rate was 86 beats per minute (bpm) with a normal rhythm. Her abdomen was flat and soft, with no tenderness or rebound pain.

**Figure 1:**
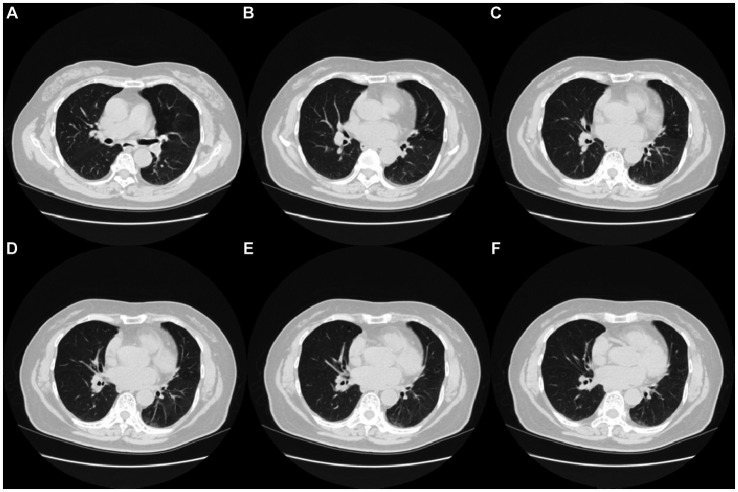
CT image results in lung infection in the patient **(A–F)** CT images of lung infection (Date 27-March-2023).

Fingertip blood was collected at the outpatient department on 27 March 2023, and a routine blood panel, with analyses of C-reactive protein (CRP) and serum amyloid protein (SAA) was performed, a rapid test for IgM antibody detection of common respiratory pathogens (Colloidal gold immune layer, Innovita, Beijing) was positive for influenza A virus, influenza B, RSV, and Cpn (Table 1). This assay kit employs the principle of immune capture for the detection of IgM antibodies against Influenza A/B, RSV, and Cpn. The testing procedure involves the collection of 10 μL of whole blood using a pipette, which is then added to the sample wells, followed by the addition of 90 μL of specimen diluent into the same sample wells, and the results are to be interpreted within a 15 to 25-min window. Upon consulting the test kit’s manual and comparing it with existing diagnostic kits in the market, in a study involving 1,512 cases, the positive concordance rate for FluA-IgM antibodies was found to be 96.88%, the negative concordance rate was 99.64%, and the overall concordance rate was 99.40%. For FluB-IgM antibodies, the rates were 97.2%, 99.63%, and 99.47%, respectively. RSV-IgM antibody exhibited rates of 97.10%, 99.71%, and 99.47%, while Cpn-antibody showed rates of 97.08%, 99.71%, and 99.47%, respectively (data from Innovita, Beijing, unpublished). Computed tomography (CT) revealed increased hazy lung markings, bronchial wall thickening, and multiple scattered spot (plaque-like) shadows in both lungs (Figure 1).

**Table 1:** Results of routine blood panel, CRP, SAA, and antibody detection of common respiratory pathogens.

Detection item (27 March 2023)	Detection result
Complete blood cell count	White blood cell 6.1 × 10^9^/L; Neutrophil 3.6 × 10^9^/L; Lymphocyte 1.9 × 10^9^/L; Monocyte 0.5 × 10^9^/L; Eosinophil 0.07 × 10^9^/L; Basophil 0.03 × 10^9^/L; Neutrophil ratio 58.3%; Lymphocyte ratio 31.4%; Monocyte ratio 8.6%; Eosinophil ratio 1.2%; Basophi ratio 0.5%.
	Red blood cell 4.4 × 10^9^/L; Hemoglobin 128 g/L.
	Platelet 246 × 10^9^/L; Mean platelet volume 8.8 fL.
Recombinant Serum Amyloid A (SAA)	8.93 mg/L
C reactive protein (CRP)	1.19 mg/L
Antibody detection of influenza A/B and parainfluenza virus and quintuple detection of respiratory pathogens (Colloidal gold immune layer, Innovita, Beijing)	Influenza A virus IgM (+); Influenza B virus IgM (+); Cpn IgM (+); RSV IgM (+); *Mycoplasma pneumoniae* IgM (−); Coxsackie virus B IgM (−); adenovirus IgM (−); parainfluenza virus IgM (−).

**Figure 2:**
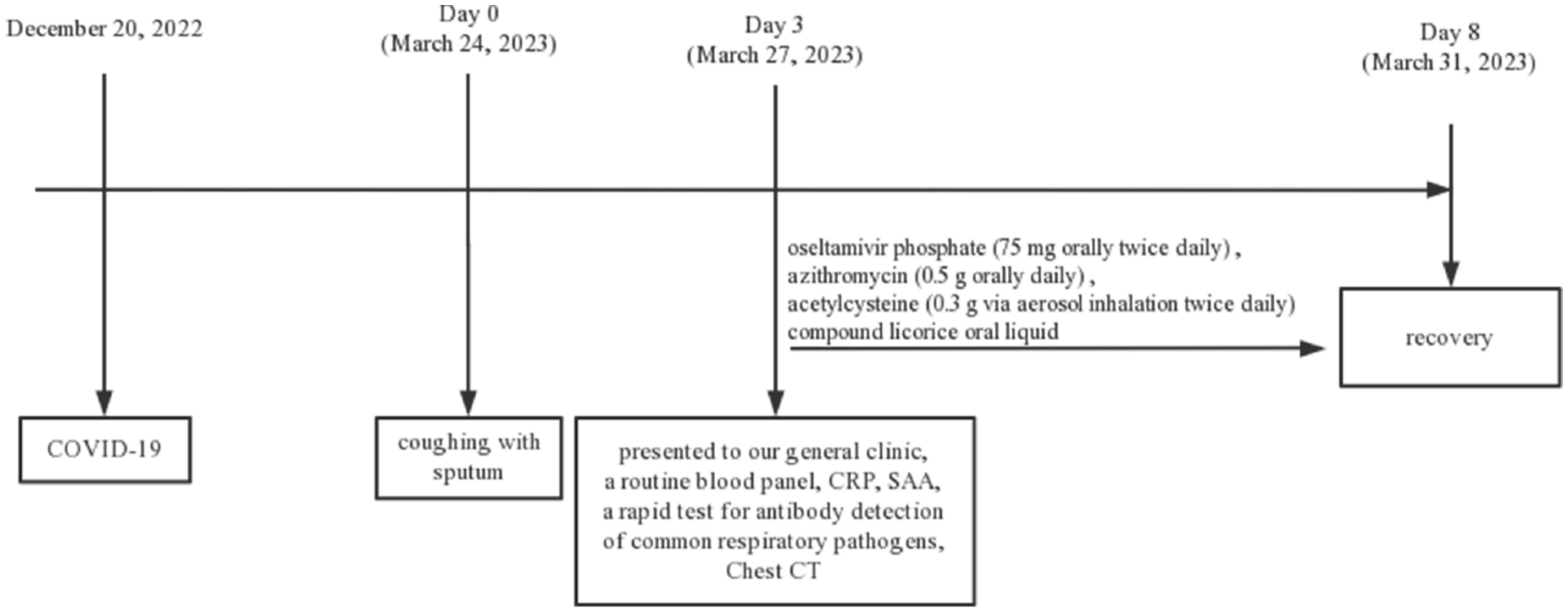
Timeline diagram of the disease course.

Due to the absence of essential equipment in our outpatient laboratory, lower respiratory tract specimens were not obtained for viral or bacterial culture, and no pathogen-specific nucleic acid testing was conducted to provide further clarification regarding the presence of co-infection. Nonetheless, a presumptive diagnosis was made, indicating potential co-infection with influenza type A, influenza B, RSV, and Cpn. We initiated immediate drug treatment for the patient, including oral oseltamivir phosphate (75 mg twice daily) as an antiviral, azithromycin (0.5 g orally daily) as an antibiotic, acetylcysteine (0.3 g via aerosol inhalation twice daily), and compound licorice oral liquid for cough relief. Because the patient refused to transfer to a superior hospital for further examination, we maintained close communication and followed up on the changes in the patient’s condition. On the eighth day, the patient had substantially reduced cough and sputum. Following treatment, her shortness of breath improved, and she achieved a complete cure with the aforementioned regimen. On April 9, during the follow-up, the patient had fully recovered, expressed satisfaction with the treatment, and declined further visits. The medical timeline is listed in Figure 2.

## 3 Literature review

**Figure 3:**
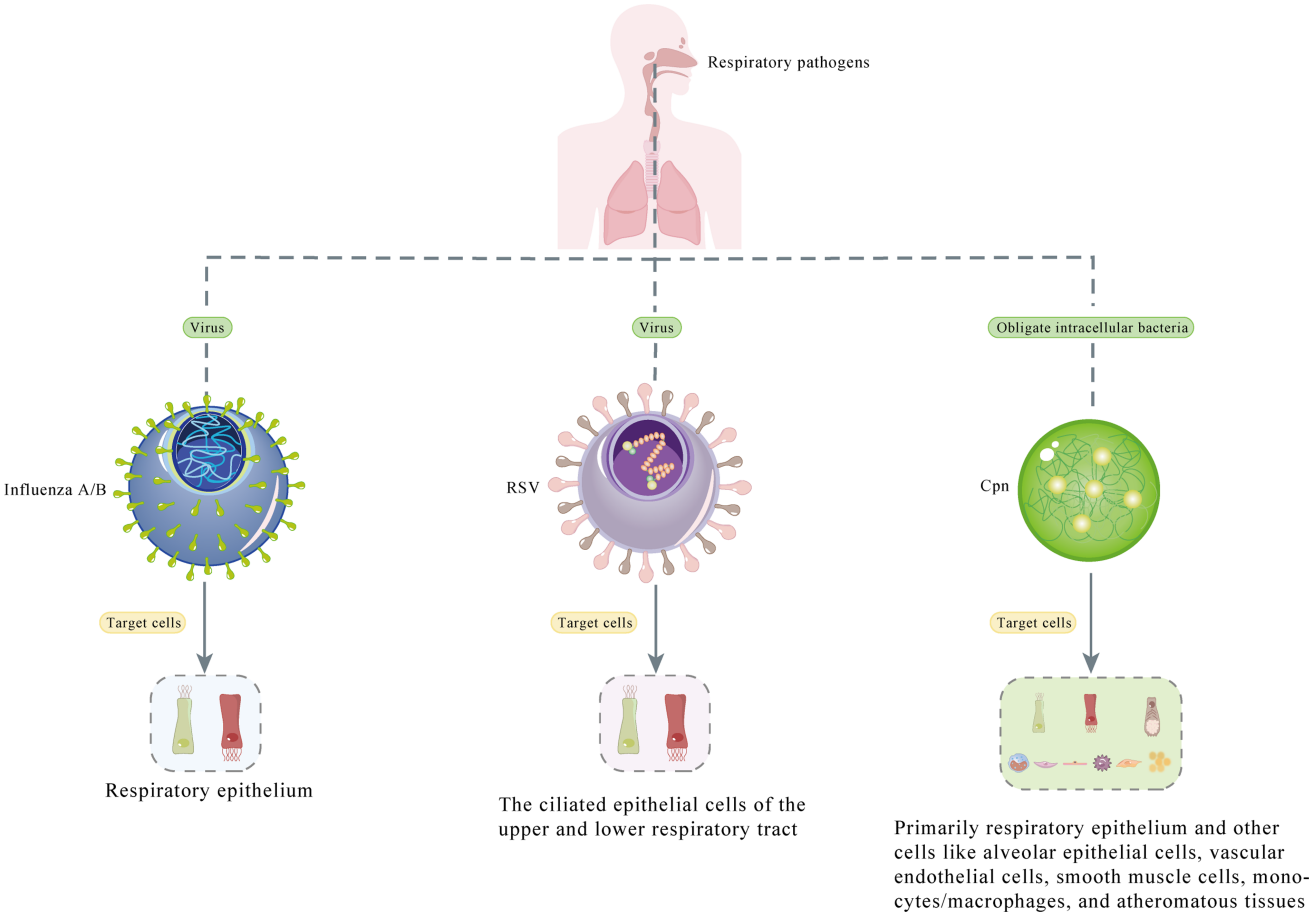
Diagram depicts the different types of pathogens (virus/bacteria) and the cells they target.

The case of co-infection with influenza A, influenza B, RSV, and Cpn presented in this paper is one of the few ever reported cases (7–20). Because the patient visited the hospital during the influenza season, was older, and had an obvious cough productive of sputum, respiratory pathogen screening and lung CT examination were performed. The precise etiology and the prognosis of the co-infection remains unclear. When genetically distinct influenza viruses of the same type co-infect the same host, there is a potential for rearrangement of viral genomic segments, leading to viral recombination, and this phenomenon should be closely monitored for surveillance and pandemic preparedness (7). Thus, We have presented a figure showing the different type of pathogens (virus/bacteria) and the cells they target (Figure 3), and conduct a comprehensive search for relevant English-language studies on PubMed to enhance our understanding of this occurrence. Table 2 presents cases of co-infection with different respiratory pathogens that were included in our review, which comprised a total of 3,077 cases (the cumulative count of all co-infected cases from 14 studies). To the best of our knowledge, there are almost no cases exhibiting co-infection with all four pathogens, as detailed in our report.

**Table 2:** Characteristics of the cases of co-infection documented in the literature.

References	*N*	Region	Age	Sex (female/total)	Co-infection of respiratory pathogens^*^	Diagnostic test	Underlying disease	Clinical presentation	Hospitalized	Died
Gregianini et al. (7)	17	Brazil	0.2–89 years	11/17	IAV + IBV	RT-PCR	7/17	Influenza-like illness	12/17	3
Mathur et al. (8)	1	America	NR	N	IAV + RSV	Virus isolation syncytial and serology	NR	Pneumonia.	NR	NR
Nickbakhsh et al. (9)	1,086	England	0–91 years	463/1,086	964 dual infections	RT-PCR	NR	NR	NR	NR
					105 triple infections					
					15 four viruses’ infection					
					2 five viruses’ infection					
Goka et al. (10)	1,214	England	1,014 Age < 5 years	502/1,014	15 IBV + RSV	RT-PCR	NR	NR	1,049/1,214	18
Perez-Garcia et al. (11)	10	Spain	3–87 years	5/10	IAV+ IBV	RT-PCR and/or cell culture	9/10	NR	NR	3
Loevinsohn et al. (12)	64	Zambia	0–51 years	NR	NR	RT-PCR	43/64	Influenza-like illness	NR	NR
Kaaijk et al. (13)	33	Netherlands	60–94 years	NR	5 influenza viruses + RSV; 28 dual-infection (NR)	RT-PCR	NR	Influenza-like illness	0/33	0
Goka et al. (14)	168	England	145 age < 5 years	87/168	dual-infection (IAV + RV/RSV/ADV/IBV/hMPV)	RT-PCR	NR	NR	123/168	16
			23 age > 5 years							
Antalis et al. (15)	68	Greece	25 age < 18 years	33/68	45 RSV + influenza virus; 23 NR	RT-PCR	11/68	Influenza-like illness	9/68	0
			43 age > 18 years							
Peci et al. (16)	156	Canada	0–88 years	89/156	100 IAV + RSV; 4 IAV + EV + RV + RSV; 1 IAV + RSV + PIV	RT-PCR	39/156	Influenza-like illness	NR	NR
Cui et al. (17)	74	China	63 age < 5 years	NR	39 influenza viruses +RSV; 2 influenza viruses + RSV + MPV	RT-PCR	NR	Influenza-like illness	62/74	NR
Zhang et al. (18)	19	China	42–85 years	9/19	12 IAV + RSV; 7 IBV + RSV	Nucleic acid detection kit	18/19	NR	19/19	7
Luchsinger et al. (19)	60	Chile	NR	NR	4 Cpn + virus	Sputum and blood cultures; IgG/IgM serology and PCR	NR	NR	NR	NR
					6 Cpn + virus + other bacteria					
					8 RSV + other viruses					
					5 influenza viruses + other viruses					
Reinton et al. (20)	107	Norway	NR	NR	1 Cpn + influenza virus	NAAT	NR	NR	NR	NR

NR, not reported; N, number; RT-PCR, Quantitative Reverse Transcription Polymerase Chain Reaction; NATT, nasopharyngeal swabs analyzed by nucleic acid amplification testing; IFV, influenza virus; IAV, influenza A virus; IVB, influenza B virus; RV, rhinovirus; RSV, respiratory syncytial virus; AdV, adenovirus; EV, enterovirus; MPV, metapneumovirus; Cpn, *Chlamydia pneumoniae*; Mpn, *Mycoplasma pneumoniae*; Bp, *Bordetella pertussis*; PIV, parainfluenza virus; CoV, coronavirus. Influenza-like illness (fever and/or cough) and/or other symptoms suggestive of respiratory infection (e.g., nasal congestion, rhinorrhea, and sore throat). ^*^Only co-infections with the four respiratory pathogens associated with this study are listed in the table.

## 4 Discussion

### 4.1 Characteristics of the four pathogens

Influenza and pneumonia are among the top 10 causes of death worldwide (21). High fever (≥38°C) and cough are common initial symptoms, followed by lower respiratory symptoms such as dyspnea, severe pneumonia, hypoxic respiratory failure, and septic shock. In some cases, secondary bacterial and fungal infections, including acute pulmonary aspergillosis (APA), can further complicate the clinical presentation as APA, a severe respiratory condition caused by Aspergillus fungi, is particularly concerning in patients with weakened immune systems or those suffering from severe influenza or pneumonia (22, 23). In particular, the spread of influenza A/B viruses causes seasonal epidemics, which are estimated to result in about 3 to 5 million cases of severe disease and about 290,000 to 650,000 fatal cases worldwide (24, 25). Influenza virus spreads through three primary mechanisms: (1) Droplet transmission: When an infected person coughs or sneezes, tiny droplets containing the virus are expelled into the air and can travel distances of up to 1 meter. Individuals in close proximity who inhale these droplets can become infected; (2) Contact transmission: Influenza virus can also be transmitted when virus-contaminated hands come into contact with the mouth, nose, or eyes; and (3) Small particle droplet nuclei (aerosols): These are small airborne particles with an aerodynamic diameter below 5 μm. They can remain suspended in the air for extended periods, but their survival depends on environmental conditions. Importantly, these aerosols can retain infectivity (26). Aerosol transmission is responsible for approximately half of all transmission events (27), and respiratory transmission through aerosols, which may contain virus particles, can occur during activities such as coughing, sneezing, talking, singing, or even normal breathing (28). Pregnant women, children under 59 months of age, older adults, people with chronic medical conditions, and people with immunosuppression are at greater risk of severe illness or complications following infection. Influenza A is more common in older people, whereas younger age groups are more susceptible to influenza B (29, 30).

RSV, a leading cause of respiratory diseases worldwide, is an enveloped, single-stranded, negative-sense RNA virus that is transmitted primarily by droplets (31). It is a major pathogen contributing to the early stages of respiratory infection in the pediatric population (32–34). Compared with influenza, RSV transmission typically has more persistent annual epidemics that occur from late fall to early spring (35, 36), and the annual incidence of RSV can reach twice that of influenza A owing to the high rate of influenza vaccination (3). RSV is the leading cause of respiratory illness in adults requiring long-term care or adult day care, as well as those living in community dwellings, especially those who are old in age and chronically ill (8, 37, 38). Additionally, respiratory and circulatory deaths in individuals aged 65 years or older were associated with RSV infection (39, 40).

Cpn is one of the most common respiratory pathogens in children and adults. It is an obligate intracellular bacterial pathogen that is mainly transmitted among individuals through respiratory droplets (6). Cpn transmission is facilitated by the ability of the pathogen to survive in aerosols in humid environments (41). Transmission through asymptomatic carriers has also been reported, with an incubation period longer than that of most respiratory infections (42). Cpn is highly prevalent in different age groups worldwide (43). However, the spread of this organism has been slow (42). Approximately 70% of respiratory infections caused by Cpn are asymptomatic or present with only mild symptoms, while the remaining 30% may progress to severe respiratory diseases, such as community-acquired pneumonia with atypical symptoms, chronic bronchitis and upper respiratory tract infections (44). This progression is particularly observed in individuals with compromised immune systems and impaired cardiopulmonary functions, in whom Cpn infection may take a severe course, potentially leading to respiratory failure and, in extreme cases, death (45). Although mortality resulting from Cpn infection can reach as high as 9.8% (46), early treatment can effectively improve prognosis (47). Furthermore, the detection of Cpn DNA in atherosclerotic plaques (AS plaques), nerve tissue, and synovial tissue substantiates the link between Cpn infection and chronic diseases such as atherosclerosis, Alzheimer’s disease, and reactive arthritis (48).

### 4.2 The definition, mechanism, and clinical features of mixed infection

The patient in this report was in good health and rarely visited the hospital, her range of daily activities was mainly community living. Her family denied feeling discomfort and refused further respiratory pathogen testing. Therefore, it was almost impossible to determine whether her family members were asymptomatic and to trace the source of her mixed infection. Notably, despite infection with four different respiratory pathogens, the patient’s clinical signs and symptoms were mild and she had a good prognosis. We searched a large body of literature to identify similar cases of mixed infections and to help improve our understanding of this phenomenon.

Respiratory virus co-infection is defined as the detection of more than one viral pathogen in the same sample. Studies have shown that co-infection with more than one virus accounts for approximately 10%–11% of all respiratory viral infections and is more common in children (9, 10). The prevalence of co-infection with various influenza viruses is minimal, and its occurrence is influenced by multiple factors (7, 9), including nosocomial transmission, the common circulation of different viruses, and host characteristics such as immunosuppression and underlying heart disease (7, 11, 49). These factors are all significantly associated with the incidence of co-infection. Additionally, the interactions between influenza A and B viruses remain unclear, potentially exhibiting either synergistic or antagonistic effects. Malausse et al. (50) indicated that influenza A can intensify influenza B infection regardless of whether the influenza A infection occurs before, concurrently with, or after influenza B infection. The inhibitory effect of influenza B on influenza A, however, is contingent upon the sequence and duration of the time interval between infections (50, 51). This finding is particularly relevant to our present case report, considering that the patient was co-infected with both influenza A and B. Even though the patient’s symptoms were mild, the potential interaction between the two influenza viruses might have influenced the clinical presentation and progression of the disease. Our findings add to current knowledge by presenting an uncommon instance of a patient with potential co-infection involving these four pathogens, while the findings from Malausse et al. provide a deeper understanding of the interactions between two of these pathogens. Together, these information enhance our understanding of respiratory virus co-infections.

Co-infection with influenza A and B has been reported previously. Gregianini et al. (7) analyzed 34,459 patients in Brazil from 2009 to 2018 and reported that among 17 patients with dual influenza A and B virus infections, the youngest was 2 months old and the oldest was 89-year-old. Notably, three patients died, all of whom had underlying heart disease, whereas a 59-year-old man with a history of pneumopathy, cardiopathy, nephropathy and hypertension infected with influenza B and two different subtypes of influenza A had a good outcome. Perez-Garcia et al. (11) reported their analysis of 10 cases of influenza A and B infections, among whom only a 65-year-old woman who had a history of heart and renal failure died. In addition, Perez-Garcia provided a summary of findings from 45 cases of co-infection involving influenza A and B and concluded that co-infection did not increase clinical severity among older adults, which aligns with the observations of several other studies (12, 13, 15, 52). However, Goka et al. (14) reported a contrasting outcome, indicating that co-infection between seasonal influenza A and influenza B viruses correlated significantly with an elevated risk of ICU admission or mortality. Thus, further investigations are warranted to clarify the clinical implications of co-infection.

Co-infection with influenza and RSV is relatively common in children and adults under the age of 30 and less common in older adults (16). It has been suggested that early circulation of influenza viruses can delay RSV epidemics (53, 54). Influenza virus infection appears to reduce the risk of co-infection with other viruses, possibly due to viral interference (13, 50, 55). Once a person is infected with the first virus, their innate and adaptive immune responses trigger a state of rapid immune activation designed to prevent co-infection with other viruses, resulting in a temporary “antiviral state” (56). Mathur et al. reported a case of influenza A and RSV co-infection that eventually developed into pneumonia (8). Falsey et al. reported nine cases involving simultaneous infection with RSV and influenza A, and one case was co-infected with RSV and influenza B. Goka et al. (10) retrospectively analyzed 30,975 samples from patients aged 0–105 years, and found that 10.3% of the samples had mixed infections, of which 0.05% were infected with influenza B and RSV, and concluded that co-infection was associated with an increased risk of admission to a general ward. However, none of the articles described the characteristics or outcomes of patients co-infected with influenza virus and RSV. Cui et al. (17) documented 39 cases (0.25%) of co-infection involving influenza and RSV in nine Chinese provinces from year 2009 to 2021. Among these cases, 10 individuals developed pneumonia, with the majority being children under 5 years old, and the authors suspecting that this phenomenon might be associated with the underdeveloped immune function observed in children. Moreover, they suggested that RSV and influenza virus co-infection was the most common type of infection. Zhang et al. (18) retrospectively analyzed 922 hospitalized adult patients with acute respiratory tract infection at the China-Japan Friendship Hospital in Beijing from January 2017 to June 2019 and found 12 patients with influenza A and RSV and 7 patients with influenza B and RSV. The average age of the patients was 64.7 years, and 9 patients were admitted to the intensive care unit (ICU), where 7 patients eventually died. One patient without any underlying disease was a 54-year-old woman (influenza B + RSV), but the final outcome was death. Although this study indicates that co-infection with influenza and RSV in adults is linked to unfavorable outcomes, it is important to note that the study had a retrospective design, was conducted in an inpatient hospital setting and reported variations in the severity, progression and timing of treatment among the cases. Thus, these factors might have influenced patient outcomes. Influenza A virus mainly infects the upper and middle respiratory tracts and causes uncomplicated influenza (57), while RSV is more likely to spread to the middle and lower respiratory tracts (58). Cellular studies have indicated the production of mixed viral particles following co-infection with influenza A virus and RSV, which possess surface glycoproteins and ribonucleoproteins from both influenza A and RSV. Notably, they utilize the RSV fusion glycoprotein to evade neutralizing antibodies against influenza A. This mechanism enables them to infect and spread among cells lacking influenza receptors, revealing a previously unknown interaction between respiratory viruses. This interaction may have implications for viral pathogenesis by expanding viral tropism and facilitating immune evasion, which may promote within-host dissemination to regions of the respiratory tract that are typically resistant to infection by one of the parent viruses, thereby potentially impacting disease pathogenesis and outcomes (59).

Viral infections often co-occur with bacterial infections, such as *Streptococcus pneumoniae* and *Staphylococcus aureus*, but co-infection with atypical bacterial pathogens like Cpn is infrequent and may be attributed to the antagonistic effects observed between the influenza virus and Cpn. These effects can be elucidated through the innate immune pathways activated by the detection of each pathogen, especially those triggered by Toll-like receptor (TLR)-2-mediated recognition (60). Notably, responses to Cpn are predominantly driven by TLR-2-mediated pathways (61), and effective clearance of influenza relies significantly on TLR-2 recognition (62). The shared aspects of innate immune recognition between influenza virus and Cpn may hinder co-infections with these pathogens and account for the observed antagonistic effects (60). Lieberman et al. (63) conducted a three-month winter prospective study of three general practices in an urban population in southern Israel to determine the etiology of respiratory infections in adults, and found 2 cases of influenza A with Cpn and 3 cases of influenza B with Cpn, with a mean age of 44.8 ± 14.2 years, all of which recovered without complications or hospitalization. A prospective study (19) including 356 patients over 60 years of age with community-acquired pneumonia in Chile from 2005 to 2007 showed that 16.9% of the patients had bacterial and viral co-infections, of which six were co-infected with Cpn, a virus, and other bacteria; however, the specific pathogens were not elaborated. This study could not determine whether the number of pathogens had an impact on the clinical outcomes. Reinton et al. (20) retrospectively analyzed 26,039 patients with community-acquired pneumonia in 2011 and found one case of influenza combined with Cpn but did not explain the basic information and outcome of the patient.

Notably, the patient had contracted SARS-CoV-2 in December 2022. Previous studies have indicated that coronaviruses are quite commonly associated with co-infections (64–66). It is noteworthy that due to the COVID-19 pandemic, the rates of positivity and seasonal patterns of various respiratory viruses have varied globally (67, 68). For instance, there has been a delayed onset of RSV outbreaks in both southern and northern hemisphere countries (69, 70), and the occurrence of seasonal influenza in China has been notably reduced (71, 72) However, an increase in RSV infections has been observed in toddlers and older children (73, 74). Taken together, it remains uncertain whether SARS-CoV-2 infection influences the occurrence of co-infections involving multiple respiratory pathogens.

Collectively, the precise outcomes of co-infections involving multiple viruses and bacteria, as well as the underlying mechanisms governing their interactions, remain unclear (75). Co-infections can lead to virus-virus interactions that affect viral replication and disease severity (55, 76). The interactions between viruses and bacteria can yield either synergistic or inhibitory effects, depending on factors such as pathogen strain, dosage and the sequence of infection (77). For example, during the later stages of respiratory syncytial virus (RSV) infection, the growth of influenza A has been found to be suppressed (78, 79). Similarly, prior Gram-positive bacterial infections have demonstrated the ability to impede influenza virus infection (80). In this specific case study, it is challenging to establish the precise sequence of infection involving the four pathogens. Therefore, we can only speculate about whether pre-existing Cpn and RSV infections may have constrained the influenza virus infection. These factors may collectively account for the mild clinical symptoms observed in our reported patient.

## 5 Conclusion

We report the case of a 72-year-old woman who was co-infected potentially with four different pathogens and present a literature review of relevant studies to help improve our understanding of this rare phenomenon. When patients visit hospitals with respiratory symptoms during the influenza season, the possibility of co-infection with more than one virus should be considered, and the possibility of co-infection with other microorganisms should not be ignored. As a general practitioner in the first line of defense, early detection and timely treatment after the onset of illness may be conducive to reducing the risk of hospitalization, complication rate, and mortality. However, further studies are needed to elucidate the clinical characteristics and outcome of co-infection.

## 6 Limitation

The main limitation of this report is that the etiological diagnosis of co-infection relied on antibody testing due to the patient’s mild symptoms and subsequent recovery. It is important to note that the patient declined further pathogen-specific nucleic acid testing and the collection of lower respiratory tract specimens for viral or bacterial culture to confirm the presence of co-infection. Although nucleic acid tests were not conducted, it is noteworthy that the antibody test used in this patient demonstrated a high level of selectivity, with an overall concordance rate of 99.4%, which supports our conclusion regarding the co-infection involving all four pathogens.

## Data availability statement

The original contributions presented in the study are included in the article/supplementary material, further inquiries can be directed to the corresponding author.

## Ethics statement

The studies involving humans were approved by Huangpu District Dapuqiao Community Health Center, Shanghai, China. The studies were conducted in accordance with the local legislation and institutional requirements. The participants provided their written informed consent to participate in this study. Written informed consent was obtained from the individual(s) for the publication of any potentially identifiable images or data included in this article.

## Author contributions

YF: Conceptualization, Visualization, Writing – original draft, Writing – review & editing. SW: Data curation, Formal analysis, Methodology, Writing – original draft, Writing – review & editing. SX: Formal analysis, Methodology, Writing – original draft, Writing – review & editing. MH: Data curation, Investigation, Methodology, Writing – original draft, Writing – review & editing. YJ: Conceptualization, Supervision, Validation, Visualization, Writing – original draft, Writing – review & editing.
